# The Influence of Atrial Fibrillation on In-Hospital Mortality in People with Hospital-Acquired Pneumonia: An Observational, Sex-Stratified Study

**DOI:** 10.3390/jcm11051179

**Published:** 2022-02-22

**Authors:** Jose M. de Miguel-Yanes, Rodrigo Jimenez-Garcia, Javier de Miguel-Diez, Valentin Hernandez-Barrera, Manuel Mendez-Bailon, Jose J. Zamorano-Leon, Ana Lopez-de-Andres

**Affiliations:** 1 Internal Medicine Department, Hospital General Universitario Gregorio Marañón, Universidad Complutense de Madrid, Instituto de Investigación Sanitaria Gregorio Marañón (IiSGM), 28007 Madrid, Spain; josemaria.demiguel@salud.madrid.org; 2 Department of Public Health & Maternal and Child Health, Faculty of Medicine, Universidad Complutense de Madrid, IdISSC, 28040 Madrid, Spain; josejzam@ucm.es (J.J.Z.-L.); anailo04@ucm.es (A.L.-d.-A.); 3 Respiratory Department, Hospital General Universitario Gregorio Marañón, Instituto de Investigación Sanitaria Gregorio Marañón (IiSGM), Universidad Complutense de Madrid, 28007 Madrid, Spain; javier.miguel@salud.madrid.org; 4 Preventive Medicine and Public Health Teaching and Research Unit, Health Sciences Faculty, Universidad Rey Juan Carlos, Alcorcón, 28032 Madrid, Spain; valentin.hernandez@urjc.es; 5 Internal Medicine Department, Hospital Universitario Clínico San Carlos, Universidad Complutense de Madrid, 28040 Madrid, Spain; manuel.mendez@salud.madrid.org

**Keywords:** atrial fibrillation, hospital-acquired pneumonia, sex differences, in-hospital mortality

## Abstract

(1) Background: The study aimed to analyze the influence of atrial fibrillation (AF) prior to hospital admission (“prevalent”) and new-onset AF diagnosed during hospital admission (“incident”) on in-hospital mortality (IHM) in women and men who developed hospital-acquired pneumonia (HAP) in Spain (2016–2019). (2) Methods: We used the Spanish Register of Specialized Care-Basic Minimum Database. (3) Results: We analyzed 38,814 cases of HAP (34.6% women; 13.5% ventilator-associated). Prevalent AF was coded in 19.9% (*n* = 7742), and incident AF in 5.5% (*n* = 2136) of HAP. Crude IHM was significantly higher for prevalent AF (34.22% vs. 27.35%, *p* < 0.001) and for incident AF (35.81% vs. 28.31%, *p* < 0.001) compared to no AF. After propensity score matching, IHM among women and men with prevalent AF was higher than among women and men with no AF (among women, 32.89% vs. 30.11%, *p* = 0.021; among men, 35.05% vs. 32.46%, *p* = 0.008). Similarly, IHM among women and men with incident AF was higher than among women and men with no AF (among women, 36.23% vs. 29.90%, *p* = 0.013; among men, 35.62% vs. 30.47%; *p* = 0.003). Sex was associated with a higher IHM only in people with incident AF (for female, OR = 1.21; 95% CI: 1.01–1.57). (4) Conclusions: Both prevalent and incident AF were associated with higher IHM in people who developed HAP. Female sex was associated with a higher IHM in incident AF.

## 1. Introduction

Hospital-acquired pneumonia (HAP) is the second leading cause of nosocomial infection [1]. Important efforts have been made to reduce its morbimortality and associated health costs, and these have been mainly successful in the case of ventilator-associated pneumonia (VAP) [2]. However, VAP only represents a small fraction of all HAPs [3]. Some experts have claimed that there is a need to implement strategies to reduce the impact of not ventilator-associated HAP (NV-HAP), which continues to be an important cause of mortality even in fully developed countries [4].

The onset of arrhythmias during hospital admission for pneumonia is a well-known complication and has been reported to be around 4–9% [5,6]. Atrial fibrillation (AF) may be a risk factor for the development of HAP due to hemodynamic changes that can affect the lungs [7]. Moreover, older research had formerly found an association between incident AF and in-hospital mortality (IHM) in people admitted for pneumonia [8]. Additional research work by Ruiz, et al., has found an association between both AF present at hospital admission that persisted during the hospital stay and new-onset AF with IHM in patients admitted for pneumococcal pneumonia [9]. Therefore, it is not senseless to think that AF could be an important factor to determine the outcome of HAP.

There are conflicting reports regarding the impact of sex in HAP [10,11]. Although cultural, behavioral, and socio-economic differences may be important determinants to explain the effect exerted by sex on the clinical management and outcomes of pneumonia [12], the hypothesis of a sex gap due to biological differences is a serious concern [3]. A deeper understanding of the complex interplay between sex, AF and hospital-acquired pneumonia could inform physicians to improve the clinical care of the patients affected by these conditions.

Here we aimed to compare the clinical characteristics and in-hospital outcomes for women and men who developed HAP during the extended period 2016–2019 in Spain according to the presence of AF prior to hospital admission and new-onset AF during the hospitalization period. We used propensity score matching (PSM) with the purpose of attenuating baseline differences for the comparisons. We finally sought the variables associated with IHM among patients coded for HAP with AF prior to hospital admission or new-onset AF during the hospitalization period according to sex.

## 2. Materials and Methods

### 2.1. Study Population

We collected data from the Spanish population older than 17 years for the 4-year period 2016–2019. We evaluated each episode coded as HAP in the Spanish Register of Specialized Care-Basic Minimum Database (RAE-CMBD). Additional details on the RAE-CMBD can be found online [13]. Only patients with a “not present on admission” indicator coded who had a hospitalization longer than 48 h were considered to meet the inclusion criteria. The codification of discharge diagnoses and therapeutic procedures was carried out according to the International Classification of Disease, Tenth Revision (ICD-10). The codes used to identify patients hospitalized with HAP (cases of VAP or NV-HAP) are defined in Table S1.

The initial number of patients with HAP identified was 39,283. Those records with missing data for age (*n* = 9; 0.02%), sex (*n* = 17; 0.01%), or duration of the hospitalization (*n* = 343; 0.88%) were excluded with no imputation of missing data. We stratified the study population according to sex, in a similar fashion to previous research from our group [14].

### 2.2. Study Variables

We sought AF codes (ICD10-codes 148.xx) among people who developed any of the two types of HAP. Each discharge diagnosis has a “present on admission (POA)” indicator assigned according to the ICD-10-CM Official Guidelines for Coding and Reporting (https://icdlist.com/icd-10/guidelines/ accessed on 4 January 2022). The reporting options and definitions for POA are “Y” (present at admission); “*n*” (not present at admission); “U” (lack documentation to determine presence at admission); “W” (provider is unable to clinically determine if the condition was present); and unreported/not used. Based on the POA indicator, we were able to discriminate between patients who had been diagnosed with AF before the index hospitalization and patients who had a new-onset episode of AF during the hospitalization period.

We assessed comorbidity with the Charlson comorbidity index (CCI) extracted with the methods for ICD-10-coded administrative databases [15]. We collected information on age, comorbidities, use of oxygen prior to the index hospitalization, and a full set of diagnostic and therapeutic procedures (e.g., bronchial fibroscopy, non-invasive lung ventilation, surgery, or dialysis) (Table S1). The main outcomes were IHM, and length of hospital stay.

### 2.3. Propensity Score Matching

To control the effect of confounding covariates when patients with and without AF were compared, we matched the study cohorts using PSM. This method is used so study subpopulations are more comparable across all observed baseline covariates [16]. We matched each woman who had a code for AF prior to hospital admission with another woman of the same age and baseline clinical conditions with no AF, and we proceeded in a similar way for AF diagnosed during hospital admission. We adhered to the same criteria for the matching process among men. The variables included in the PSM model were age and all the clinical conditions analyzed. Multivariable logistic regression models were constructed to estimate the PS for each individual. The matching method chosen was one-to-one using calipers of width equal to 0.2 of the standard deviation of the logit of the PS. To assess the quality of the samples after PSM, we estimated the absolute standardized difference before and after matching for each of the four matchings conducted. Shown in Supplementary Figures S1–S4 are the Love Plots showing the absolute standardized differences before and after PSM. As can be seen in these figures, none of the absolute standardized differences after PSM were above 10%, which would indicate noteworthy imbalance [16].

### 2.4. Statistical Analysis

As descriptive statistics for continuously distributed variables, we reported means with standard deviations (SDs) or medians with interquartile ranges (IQRs), and for categorical variables, absolute frequencies, and proportions. Using the t test or the Mann–Whitney test, we compared the continuously distributed variables, and with the chi-square test, we compared the categorical variables. We used McNemar’s test and a paired t test to compare the study subgroups after PSM [16].

We used multivariable logistic regression analyses to identify the variables independently associated with IHM. As proposed by Hosmer et al., we constructed models separately for men and women [17]. We analyzed the effect of sex in two models: (1) all patients (before PSM) with AF prior to hospital admission; and (2) all patients (before PSM) with AF diagnosed during the hospitalization period. The results were expressed as odds ratios (ORs) with their 95% confidence intervals (95% CIs).

The software used for matching and the statistical analysis was Stata version 14 (Stata, College Station, TX, USA). We set statistical significance at a two-sided *p*-value of <0.05, with no correction for multiple statistical tests, even though this can lead to inflation of the type I statistical error.

### 2.5. Ethical Aspects

The RAE-CMBD is owned by the Spanish Ministry of Health and can be accessed upon request [18]. This registry is anonymized and under public access, which means that according to Spanish legislation, approval by an ethics committee can be waived.

## 3. Results

### 3.1. Clinical Characteristics and In-Hospital Outcomes for the Overall Population According to AF Prior to Hospital Admission Status

We collected information on 38,814 cases of HAP (33,588 cases of NV-HAP (86.5%) plus 5226 cases of VAP) in people ≥ 18 years old (Table S2), out of which 13,432 cases corresponded to women (34.6%). Overall, people coded for AF prior to hospital admission represented 19.9% (*n* = 7742) of the population. Women constituted 38.6% of the population with prior history of AF, whilst the proportion of women in the population with no history of AF was 31.8%.

People with AF prior to hospital admission were older than people with no history of AF (79.06 ± 9.73 vs. 67.99 ± 15.97 years; *p* < 0.001) and had more comorbidities (*p* < 0.001). They more commonly suffered from cardiovascular conditions, dementia, chronic respiratory disease, type 2 diabetes mellitus, renal disease, and used oxygen at home, but less frequently had liver disease or cancer (all *p* values < 0.001) (Table S2). People with AF prior to hospital admission less often underwent surgery, bronchial fibroscopy, dialysis, and non-invasive lung ventilation during admission than people with no prior AF (all *p* values < 0.001) (Table S2). Length of hospital stay was lower for people with previous history of AF (21 ± 22 vs. 25 ± 29 days), yet crude IHM was significantly higher in people with history of AF prior to hospital admission (34.22% vs. 27.35%; *p* < 0.001).

### 3.2. Clinical Characteristics and In-Hospital Outcomes for the Overall Population According to AF Diagnosed during the Hospitalization Period Status

People with new-onset of AF during hospital admission represented 5.5% (*n* = 2136) of the total population. The proportion of women with AF diagnosed during hospital admission was 31.8%, vs. 34.8% in the population who did not experience new-onset AF.

People with AF diagnosed during hospital admission were older than people without AF (72.54 ± 11.35 vs. 70.06 ± 15.77 years; *p* < 0.001) and had more comorbidities (*p* < 0.001). They more commonly suffered from chronic myocardial infarction and heart failure (*p* values < 0.001), but less frequently had dementia (*p* < 0.001), type 2 diabetes mellitus (*p* = 0.002), liver disease (*p* = 0.028), or used oxygen at home (*p* = 0.001) (Table S2). People in whom AF was diagnosed during hospital admission more often underwent surgery, bronchial fibroscopy, dialysis, and non-invasive lung ventilation than people without AF (all *p* values ≤ 0.001) (Table S2). Both length of hospital stay (31 ± 35 vs. 23 ± 27 days), and crude IHM (35.81% vs. 28.31%; *p* < 0.001) were higher in people with AF diagnosed during the hospitalization period than in people without AF.

### 3.3. Clinical Characteristics and In-Hospital Outcomes for Women and Men by AF Prior to Hospital Admission Status after Propensity Score Matching

After PSM, women with AF prior to hospital admission were slightly younger (81.28 ± 9.15 vs. 82.02 ± 9.28; *p* = 0.002), but more often suffered from heart failure (*p* = 0.04) and used oxygen at home (*p* = 0.003). Contrarily, they less frequently suffered from dementia (*p* < 0.001) and less often underwent surgery (*p* = 0.02) (Table 1). Whereas length of hospital stay did not differ between both groups, IHM remained higher in women who had AF before hospital admission (32.89% vs. 30.11%; *p* = 0.021).

After PSM, men with AF prior to hospital admission were slightly younger (77.67 ± 9.81 vs. 78.20 ± 9.79; *p* = 0.008) and less frequently had dementia (*p* = 0.001), but more often suffered from heart failure (*p* < 0.013) and received non-invasive lung ventilation (*p* = 0.013) (Table 2). Again, although length of hospital stay did not differ between both groups, IHM remained higher in men who had AF before hospital admission (35.05% vs. 32.46%; *p* = 0.008).

### 3.4. Clinical Characteristics and In-Hospital Outcomes for Women and Men by AF Diagnosed during the Hospitalization Period Status after Propensity Score Matching

After PSM, women with AF diagnosed during the hospitalization period more often received non-invasive lung ventilation and dialysis (both *p* < 0.001) (Table 3). Both length of hospital stay (29 ± 32 vs. 22 ± 27 days; *p* = 0.004) and IHM (36.23% vs. 29.90%; *p* = 0.013) were higher than among women with no onset of AF during the hospitalization period.

After PSM, men with AF diagnosed during the hospitalization period less commonly had dementia (*p* = 0.03) or used oxygen at home (*p* = 0.012), but more often received non-invasive lung ventilation and dialysis (both *p* < 0.001) (Table 4). Both length of hospital stay (32 ± 38 vs. 25 ± 30 days; *p* < 0.001) and IHM (35.62% vs. 30.47%; *p* = 0.003) were significantly higher than among men with no onset of AF during the hospitalization period.

### 3.5. Multivariable Analysis of Factors Associated with In-Hospital Mortality during Admission for HAP among Patients with AF Prior to Hospital Admission

Shown in Table S3 are the results of the multivariable logistic regression models conducted with the entire database of patients with AF prior to hospital admission who developed HAP according to sex, to assess variables associated with IHM. The risk of dying during hospital admission for HAP among patients with AF prior to hospital admission increased in both sexes with advanced age, cerebrovascular disease (OR = 1.31 (95% CI: 1.15–1.50)) and cancer (OR = 1.57 (95% CI: 1.37–1.80)) (Table S3). Albeit undergoing surgery was associated with a lower IHM (OR = 0.72; 95% CI: 0.64–0.81), dialysis (OR = 2.18; 95% CI: 1.75–2.72), non-invasive lung ventilation (OR = 3.07; 95% CI: 2.63–3.57), and VAP (OR = 1.29; 95% CI: 1.05–1.58) were associated with a higher IHM (ORs and 95% CIs shown above are for the overall population). Sex was not associated with a higher IHM.

### 3.6. Multivariable Analysis of Factors Associated with In-Hospital Mortality during Admission for HAP among Patients with AF Diagnosed during the Hospitalization Period

As can be seen in Table S4, when the entire population of patients with AF diagnosed during the hospitalization period who suffered HAP was analyzed with multivariable logistic regression to identify predictors of IHM, we found that advanced age increased mortality only among men (Table S4). Chronic respiratory disease (OR = 1.45; 95% CI: 1.08–1.89), cancer (OR = 1.85; 95% CI: 1.47–2.32), dialysis (OR = 2.31; 95% CI: 1.78–3.01), non-invasive lung ventilation (OR = 1.96; 95% CI: 1.57–2.44), and VAP (OR = 2.45; 95% CI: 1.44–4.07) were associated with a higher IHM (ORs and 95% CIs shown above are for the overall population). Female sex was associated with a higher IHM in people who developed AF during the hospitalization period (OR = 1.21; 95% CI: 1.01–1.57).

## 4. Discussion

In this study, we found that most cases of HAP were NV-HAP (86.5%), according to previous reports [19]. Almost 20 percent of the people older than 17 years who developed HAP had AF prior to hospital admission, yet only around 5 percent had new-onset AF during the hospitalization period. These figures vary in the previously published literature depending on factors such as the age of the population included, the quality of the coding process, and the qualification of “present on admission”, more specifically for patients with clinical history of paroxysmal AF. Our definition of new-onset AF was restrictive, and may have conditioned lower incidence rates for new-onset AF in this group of very ill patients with high mortality rates. Indeed, we discarded those patients who presented at the ED with sinus rhythm but had previously had AF in its paroxysmal form and were therefore coded as “present on admission”. Not surprisingly, these are the patients who most often present episodes of AF when suffering from pneumonia [6]. In subpopulations of patients admitted to the intensive care unit for pneumonia, these numbers have been reported to be higher [20]. Several mechanisms for the new onset of AF in this context have been proposed, including hypoxia, heart scarring, or rises in cytosolic calcium, which affect endothelial cadherin junctions, thus inducing apoptosis that leads to cardiac injury and arrhythmia [21].

Compared with no AF, crude IHM was significantly higher, both in people with AF prior to hospital admission and people with new-onset AF diagnosed during the hospitalization period. After PSM, women and men with AF prior to hospital admission had higher adjusted IHM than women and men with no AF, despite being significantly younger. Moreover, women and men with new-onset AF diagnosed during the hospitalization period had higher IHM than women and men with no AF; they also had more prolonged hospital stays. The association between new-onset AF and mortality in severely ill patients has been described by other authors [22,23]. Nevertheless, the association between AF prior to hospital admission and IHM in pneumonia is less well established [9]. Whether new-onset AF is a marker of higher clinical severity, of distinct pathophysiologic mechanisms, is deleterious by itself, or by the therapeutic measures that its incidence calls for cannot be clarified with the design of our study.

The risk of dying during hospital admission for HAP among patients with both AF prior to hospital admission or AF diagnosed during the hospitalization period increased with age, comorbidities, interventional non-surgical procedures, including non-invasive lung ventilation, and when the index episode was a VAP. In our study, as compared with no AF, patients with AF prior to hospital admission received non-invasive lung ventilation in a quite balanced way. However, as compared with no AF, patients with new-onset AF during the hospitalization period were far more often treated with non-invasive lung ventilation. Acutely established AF may contribute to hemodynamic instability during an acute infection, probably signaling adrenergic overstimulation [24]. Perhaps a worse clinical situation during admission prompted the indication of a higher number of procedures in a population with an a priori higher probability of death during the hospital stay, or with limitation of therapeutic efforts and do-not-resuscitate orders. We must be cautious when interpreting significant associations found in observational studies where the issue of confounding by indication may be underlying. Nonetheless, these arguments are merely speculative.

We were somewhat surprised to see that having undergone surgery during the index hospitalization appeared as a protective factor for mortality in people with AF prior to hospital admission. We had previously reported mortality rates around 30% in people who developed postoperative pneumonia in Spain from 2001–2015 [25]. Arguably, both people admitted to the hospital for a surgical procedure, and people admitted to the hospital for other reasons who underwent a surgical procedure had better performance and baseline health status than people who did not undergo any surgical procedure. This may be the reason for this apparent paradox of a better outcome in operated patients. As opposed to this, patients suffering from cancer admitted for surgery might be less fit than non-cancer patients. Perhaps, patients with prevalent AF underwent a more thorough cardiac evaluation before admission that could have helped to prevent adverse outcomes. Regardless, we acknowledge that this reasoning is conjectural, since we have no clear-cut explanation for this finding.

We detected that people who developed VAP had a higher IHM than people who developed NV-HAP among both sexes, and the mortality risk associated with VAP almost doubled in the group of patients with new-onset AF during the hospitalization period compared with the mortality risk found in people with AF prior to hospital admission. This finding is in line with previous reports [26,27], and in part it is probably due to the severity and type of underlying disease leading to the indication of mechanical ventilation, whilst not being driven by the procedure itself. There is general agreement on the persistently high attributable mortality of VAP [28].

Female sex was associated with a 21% higher IHM in patients with new-onset AF triggered during their hospitalization period but was not associated with IHM in patients with AF prior to hospital admission. It is unclear why we are seeing this sex gap exclusively in the case of new-onset AF during the hospitalization period. We were able to adjust for baseline congestive heart failure, but we could not account for chronic left ventricular ejection fraction, and differences in this parameter could be confounding the results. This finding may truly reflect some heterogeneity in the impact of the onset of AF on mortality [29], a more conservative approach in the very ill women with hemodynamic instability, or contrarily, differential deleterious effects of the antiarrhythmic drugs eventually used in our clinical practice among women [30]. In Spain, there has been previously reported differences in the clinical management and outcomes of men and women with diabetes admitted to hospital with AF [31]. The sex differences in the outcomes for people with AF in the general population have been described before in epidemiology studies [32], although few subsequent studies have found similar effects for AF on mortality among both sexes [33]. For specific conditions other than pneumonia, such as heart failure, sex-related differences have been reported as well [34]. Schnabel et al. found that in a “real-world” European AF registry, women were more symptomatic but less likely to receive invasive rhythm control therapy such as electrical cardioversion or ablation. However, there were no statistically significant sex differences in 1-year stroke/transient ischemic attack/arterial thromboembolism and major bleeding events [35]. Marzona et al. conducted a meta-analysis to assess if women patients with AF could have a greater risk for stroke and thromboembolic events. These authors concluded that sex may act as a stroke risk modifier, particularly in elderly and very elderly AF subjects, conferring women a significant increase in stroke risk [36].

Despite this, there is sparse evidence on how prevalent and incident AF modify IHM in people admitted for pneumonia. We believe that this study, along with previous research from our group [3], contributes to fill a gap in the medical literature, where the complex interaction between sex and the two common conditions of AF and pneumonia deserves further attention.

As main strengths of our study, we underscore its large sample size, with data from 38,814 episodes of HAP, the widespread coverage of the Spanish population by the RAE-CMBD (>95% of all hospital admissions), and the standardized methodology. However, some limitations should be underscored. First, our data source is an administrative database built with the information that physicians keep in the discharge report and is also dependent on manual coding on behalf of the administrative staff. Second, despite a PSM process that most likely contributed to attenuate the differences in baseline characteristics and clinical variables, a total exclusion of residual confounding is difficult to achieve in observational studies. Third, detailed information on some clinical variables, such as antibiotic therapy, smoking status, obesity, trends in rhythm control, or anticoagulation therapy in these patients, which might have had an impact on cardiovascular events and mortality, were not included. Forth, according to the RAE-CMBD methodology, only those diagnoses that have induced the use of additional therapeutic or diagnosis procedures during the hospital admission or have negatively affected the length of hospital stay or the IHM, should be recorded in the secondary diagnostic fields [37]. Thus, many of these items are probably under-codified, potentially biasing the results from the data analysis, and therefore were not included. Fifth, we performed a pool analysis of cases of VAP and NV-HAP, albeit these two conditions may not share the clinical characteristics underlying each of them. For this same reason, invasive lung ventilation (always present in the case of VAP) was not included in the multivariate model. Sixth, no external validation has been performed in this study to assess the validity of the identified predictive factors using an external dataset. Lastly, anonymity did not allow the accuracy of some specific pieces of information (i.e., people who moved from one hospital to another would appear twice).

## 5. Conclusions

Our study shows an association between both AF prior to hospital admission and new-onset AF diagnosed during the hospitalization period for HAP with IHM. Whether AF is a marker of higher clinical severity or deleterious by itself needs to be elucidated. We also found that female sex was associated with a higher IHM in new-onset AF. Future research efforts should focus on identifying possible reasons that explain sex differences to ease the way to reduce them.

## Figures and Tables

**Table 1:** Distribution of study covariates and hospital outcomes for women with hospital-acquired pneumonia in Spain (2016–2019), according to the presence of AF diagnosed prior to hospital admission (POA = YES) before and after propensity score matching (PSM).

	Before PSM			After PSM		
	Atrial Fibrillation	No Atrial Fibrillation	*p*-Value	Atrial Fibrillation	No Atrial Fibrillation	*p*-Value
HAP, *n*	2986	10,446	NA	2986	2986	NA
NV-HAP, *n*, (%)	2799 (93.74)	9128 (87.38)	<0.001	2799 (93.74)	2814 (94.24)	0.414
VAP, *n*, (%)	187 (6.26)	1318 (12.62)	<0.001	187 (6.26)	172 (5.76)	0.414
Age, mean (SD)	81.28 (9.15)	69.86 (16.78)	<0.001	81.28 (9.15)	82.02 (9.28)	0.002
18–54 years old, *n* (%)	38 (1.27)	1953 (18.70)	<0.001	38 (1.27)	34 (1.14)	0.635
55–69 years old, *n* (%)	281 (9.41)	2572 (24.62)	<0.001	281 (9.41)	261 (8.74)	0.368
70–84 years old, *n* (%)	1431 (47.92)	3653 (34.97)	<0.001	1431 (47.92)	1323 (44.31)	0.005
≥85 years old, *n* (%)	1236 (41.39)	2268 (21.71)	<0.001	1236 (41.39)	1368 (45.81)	0.001
CCI, mean (SD)	2 (1.17)	1.41 (1.11)	<0.001	2 (1.17)	1.97 (1.21)	0.323
Myocardial infarction, *n* (%)	165 (5.53)	490 (4.69)	0.062	165 (5.53)	174 (5.83)	0.615
Congestive heart failure, *n* (%)	1653 (55.36)	2256 (21.60)	<0.001	1653 (55.36)	1574 (52.71)	0.040
Peripheral vascular disease, *n* (%)	161 (5.39)	469 (4.49)	0.040	161 (5.39)	168 (5.63)	0.691
Cerebrovascular disease, *n* (%)	624 (20.90)	1453 (13.91)	<0.001	624 (20.90)	614 (20.56)	0.750
Dementia, *n* (%)	226 (7.57)	650 (6.22)	0.009	226 (7.57)	310 (10.38)	<0.001
Chronic respiratory disease, *n* (%)	545 (18.25)	1487 (14.24)	<0.001	545 (18.25)	539 (18.05)	0.840
Type 2 diabetes mellitus, *n* (%)	940 (31.48)	2245 (21.49)	<0.001	940 (31.48)	951 (31.85)	0.760
Rheumatoid disease, *n* (%)	111 (3.72)	348 (3.33)	0.306	111 (3.72)	122 (4.09)	0.462
Peptic ulcer, *n* (%)	43 (1.44)	198 (1.90)	0.098	43 (1.44)	55 (1.84)	0.222
Mild/moderate/severe liver disease, *n* (%)	144 (4.82)	753 (7.21)	<0.001	144 (4.82)	130 (4.35)	0.387
Hemiplegia or paraplegia, *n* (%)	205 (6.87)	568 (5.44)	0.003	205 (6.87)	155 (5.19)	0.007
Renal disease, *n* (%)	856 (28.67)	1461 (13.99)	<0.001	856 (28.67)	802 (26.86)	0.119
Cancer and metastatic cancer, *n* (%)	285 (9.54)	2285 (21.87)	<0.001	285 (9.54)	272 (9.11)	0.563
AIDS, *n* (%)	1 (0.03)	56 (0.54)	<0.001	1 (0.03)	2 (0.07)	0.564
Underwent surgery, *n* (%)	905 (30.31)	5165 (49.44)	<0.001	905 (30.31)	989 (33.12)	0.020
Bronchial fibroscopy, *n* (%)	38 (1.27)	284 (2.72)	<0.001	38 (1.27)	33 (1.11)	0.551
Non-invasive lung ventilation, *n* (%)	391 (13.09)	2461 (23.56)	<0.001	391 (13.09)	379 (12.69)	0.643
Dialysis, *n* (%)	115 (3.85)	565 (5.41)	0.001	115 (3.85)	109 (3.65)	0.683
Oxygen prior to hospitalization, *n* (%)	137 (4.59)	224 (2.14)	<0.001	137 (4.59)	93 (3.11)	0.003
LOHS, median (IQR)	20 (20)	24 (28)	<0.001	20 (20)	20 (20)	0.300
IHM, *n* (%)	982 (32.89)	2769 (26.51)	<0.001	982 (32.89)	899 (30.11)	0.021

POA: present on admission; HAP: hospital-acquired pneumonia; NV-HAP: non-ventilator hospital-acquired pneumonia; VAP: ventilator-associated pneumonia; SD: standard deviation; CCI: Charlson comorbidity index; AIDS: acquired immune deficiency syndrome; LOHS: length of hospital stay; IQR: interquartile range; IHM: in-hospital mortality.

**Table 2:** Distribution of study covariates and hospital outcomes for men with hospital-acquired pneumonia in Spain (2016–2019), according to the presence of AF diagnosed prior to hospital admission (POA = YES) before and after propensity score matching (PSM).

	Before PSM			After PSM		
	Atrial Fibrillation	No Atrial Fibrillation	*p*-Value	Atrial Fibrillation	No Atrial Fibrillation	*p*-Value
HAP, *n*	4756	20,626	NA	4756	4756	NA
NV-HAP, *n*, (%)	4264 (89.66)	17,397 (84.35)	<0.001	4264 (89.66)	4256 (89.49)	0.788
VAP, *n*, (%)	492 (10.34)	3229 (15.65)	<0.001	492 (10.34)	500 (10.51)	0.788
Age, mean (SD)	77.67 (9.81)	67.05 (15.46)	<0.001	77.67 (9.81)	78.20 (9.79)	0.008
18–54 years old, *n* (%)	113 (2.38)	4135 (20.05)	<0.001	113 (2.38)	97 (2.04)	0.264
55–69 years old, *n* (%)	778 (16.36)	6386 (30.96)	<0.001	778 (16.36)	740 (15.56)	0.287
70–84 years old, *n* (%)	2615 (54.98)	7722 (37.44)	<0.001	2615 (54.98)	2595 (54.56)	0.680
≥85 years old, *n* (%)	1250 (26.28)	2383 (11.55)	<0.001	1250 (26.28)	1324 (27.84)	0.088
CCI, mean (SD)	2.23 (1.28)	1.63 (1.21)	<0.001	2.23(1.28)	2.21(1.29)	0.498
Myocardial infarction, *n* (%)	570(11.98)	1859(9.01)	<0.001	570(11.98)	554(11.65)	0.611
Congestive heart failure, *n* (%)	2089(43.92)	3544(17.18)	<0.001	2089 (43.92)	1969 (41.40)	0.013
Peripheral vascular disease, *n* (%)	628 (13.20)	2028 (9.83)	<0.001	628 (13.20)	630 (13.25)	0.952
Cerebrovascular disease, *n* (%)	921 (19.37)	2942 (14.26)	<0.001	921 (19.37)	913 (19.20)	0.835
Dementia, *n* (%)	244 (5.13)	776 (3.76)	<0.001	244 (5.13)	317 (6.67)	0.001
Chronic respiratory disease, *n* (%)	1526 (32.09)	4691 (22.74)	<0.001	1526 (32.09)	1532 (32.21)	0.895
Type 2 diabetes mellitus, *n* (%)	1520 (31.96)	4638 (22.49)	<0.001	1520 (31.96)	1547 (32.53)	0.554
Rheumatoid disease, *n* (%)	83 (1.75)	255 (1.24)	0.006	83 (1.75)	83 (1.75)	0.999
Peptic ulcer, *n* (%)	86 (1.81)	462 (2.24)	0.065	86 (1.81)	106 (2.23)	0.145
Mild/moderate/severe liver disease, *n* (%)	385 (8.10)	2367 (11.48)	<0.001	385 (8.10)	349 (7.34)	0.167
Hemiplegia or paraplegia, *n* (%)	282 (5.93)	1297 (6.29)	0.356	282 (5.93)	280 (5.89)	0.931
Renal disease, *n* (%)	1348 (28.34)	2936 (14.23)	<0.001	1348 (28.34)	1319 (27.73)	0.508
Cancer and metastatic cancer, *n* (%)	909 (19.11)	5656 (27.42)	<0.001	909 (19.11)	904 (19.01)	0.896
AIDS, *n* (%)	9 (0.19)	223 (1.08)	<0.001	9 (0.19)	12 (0.25)	0.512
Underwent surgery, *n* (%)	1895 (39.84)	10,987 (53.27)	<0.001	1895 (39.84)	1928 (40.54)	0.490
Bronchial fibroscopy, *n* (%)	128 (2.69)	687 (3.33)	0.024	128 (2.69)	105 (2.21)	0.127
Non-invasive lung ventilation, *n* (%)	1075 (22.60)	6338 (30.73)	<0.001	1075 (22.60)	975 (20.50)	0.013
Dialysis, *n* (%)	294 (6.18)	1571 (7.62)	0.001	294 (6.18)	340 (7.15)	0.059
Oxygen prior to hospitalization, *n* (%)	196 (4.12)	506 (2.45)	<0.001	196 (4.12)	207 (4.35)	0.576
LOHS, median (IQR)	22 (23)	25 (29)	<0.001	22 (23)	22 (23)	0.700
IHM, *n* (%)	1667 (35.05)	5729 (27.78)	<0.001	1667 (35.05)	1544 (32.46)	0.008

POA: present on admission; HAP: hospital-acquired pneumonia; NV-HAP: non-ventilator hospital-acquired pneumonia; VAP: ventilator-associated pneumonia; SD: standard deviation; CCI: Charlson comorbidity index; AIDS: acquired immune deficiency syndrome; LOHS: length of hospital stay; IQR: interquartile range; IHM: in-hospital mortality.

**Table 3:** Distribution of study covariates and hospital outcomes for women with hospital-acquired pneumonia in Spain (2016–2019), according to the presence of AF diagnosed during the hospitalization period (POA = NO) before and after propensity score matching (PSM).

	Before PSM			After PSM		
	Atrial Fibrillation	No Atrial Fibrillation	*p*-Value	Atrial Fibrillation	No Atrial FIbrillation	*p*-Value
HAP, *n*	679	12,753	NA	679	679	NA
NV-HAP, *n* (%)	566 (83.36)	11,361 (89.08)	<0.001	566 (83.36)	589 (86.75)	0.080
VAP, *n* (%)	113 (16.64)	1392 (10.92)	<0.001	113 (16.64)	90 (13.25)	0.080
Age, mean (SD)	75.91 (11.23)	72.21 (16.32)	<0.001	75.91 (11.23)	76.13 (11.90)	0.723
18–54 years old, *n* (%)	26 (3.83)	1965 (15.41)	<0.001	26 (3.83)	29 (4.27)	0.406
55–69 years old, *n* (%)	159 (23.42)	2694 (21.12)	0.155	159 (23.42)	150 (22.09)	0.932
70–84 years old, *n* (%)	336 (49.48)	4748 (37.23)	<0.001	336 (49.48)	330 (48.60)	0.057
≥ 85 years old, *n* (%)	158 (23.27)	3346 (26.24)	0.086	158 (23.27)	170 (25.04)	0.092
CCI, mean (SD)	1.66 (1.15)	1.53 (1.15)	0.005	1.66 (1.15)	1.63 (1.14)	0.602
Myocardial infarction, *n* (%)	64 (9.43)	591 (4.63)	<0.001	64 (9.43)	48 (7.07)	0.114
Congestive heart failure, *n* (%)	285 (41.97)	3624 (28.42)	<0.001	285 (41.97)	287 (42.27)	0.912
Peripheral vascular disease, *n* (%)	40 (5.89)	590 (4.63)	0.129	40 (5.89)	36 (5.30)	0.637
Cerebrovascular disease, *n* (%)	103 (15.17)	1974 (15.48)	0.828	103 (15.17)	100 (14.73)	0.819
Dementia, *n* (%)	35 (5.15)	841 (6.59)	0.139	35 (5.15)	32 (4.71)	0.707
Chronic respiratory disease, *n* (%)	110 (16.20)	1922 (15.07)	0.424	110 (16.20)	105 (15.46)	0.710
Type 2 diabetes mellitus, *n* (%)	157 (23.12)	3028 (23.74)	0.711	157 (23.12)	158 (23.27)	0.949
Rheumatoid disease, *n* (%)	19 (2.80)	440 (3.45)	0.362	19 (2.80)	26 (3.83)	0.289
Peptic ulcer, *n* (%)	12 (1.77)	229 (1.80)	0.957	12 (1.77)	18 (2.65)	0.268
Mild/moderate/severe liver disease, *n* (%)	40 (5.89)	857 (6.72)	0.399	40 (5.89)	46 (6.77)	0.504
Hemiplegia or paraplegia, *n* (%)	37 (5.45)	736 (5.77)	0.726	37 (5.45)	38 (5.60)	0.905
Renal disease, *n* (%)	108 (15.91)	2209 (17.32)	0.341	108 (15.91)	91 (13.40)	0.192
Cancer and metastatic cancer, *n* (%)	117 (17.23)	2453 (19.23)	0.196	117 (17.23)	117 (17.23)	0.999
AIDS, *n* (%)	0 (0.00)	57 (0.45)	0.081	0 (0.00)	3 (0.44)	0.083
Underwent surgery, *n* (%)	400 (58.91)	5670 (44.46)	<0.001	400 (58.91)	422 (62.15)	0.222
Bronchial fibroscopy, *n* (%)	25 (3.68)	297 (2.33)	0.025	25 (3.68)	19 (2.80)	0.358
Non-invasive lung ventilation, *n* (%)	264 (38.88)	2588 (20.29)	<0.001	264 (38.88)	161 (23.71)	<0.001
Dialysis, *n* (%)	85 (12.52)	595 (4.67)	<0.001	85 (12.52)	32 (4.71)	<0.001
Oxygen prior to hospitalization, *n* (%)	14 (2.06)	347 (2.72)	0.301	14 (2.06)	21 (3.09)	0.231
LOHS, median (IQR)	29 (32)	22 (26)	<0.001	29 (32)	22 (27)	0.004
IHM, *n* (%)	246 (36.23)	3505 (27.48)	<0.001	246 (36.23)	203 (29.90)	0.013

POA: present on admission; HAP: hospital-acquired pneumonia; NV-HAP: non-ventilator hospital-acquired pneumonia; VAP: ventilator-associated pneumonia; SD: standard deviation; CCI: Charlson comorbidity index; AIDS: acquired immune deficiency syndrome; LOHS: length of hospital stay; IQR: interquartile range; IHM: in-hospital mortality.

**Table 4:** Distribution of study covariates and hospital outcomes for men with hospital-acquired pneumonia in Spain (2016–2019), according to the presence of AF diagnosed during the hospitalization period (POA = NO) before and after propensity score matching (PSM).

	Before PSM			After PSM		
	Atrial Fibrillation	No Atrial Fibrillation	*p*-Value	Atrial Fibrillation	No Atrial Fibrillation	*p*-Value
HAP, *n*	1457	23,925	NA	1457	1457	NA
NV-HAP, *n* (%)	1133 (77.76)	20,528 (85.80)	<0.001	1133 (77.76)	1161 (79.68)	0.205
VAP, *n* (%)	324 (22.24)	3397 (14.20)	<0.001	324 (22.24)	296 (20.32)	0.205
Age, mean (SD)	70.96 (11.07)	68.92 (15.35)	<0.001	70.96 (11.07)	71.62 (11.56)	0.115
18–54 years old, *n* (%)	115 (7.89)	4133 (17.27)	<0.001	115 (7.89)	113 (7.76)	0.347
55–69 years old, *n* (%)	493 (33.84)	6671 (27.88)	<0.001	493 (33.84)	458 (31.43)	0.613
70–84 years old, *n* (%)	708 (48.59)	9629 (40.25)	<0.001	708 (48.59)	713 (48.94)	0.001
≥ 5 years old, *n* (%)	141 (9.68)	3492 (14.60)	<0.001	141 (9.68)	173 (11.87)	<0.001
CCI, mean (SD)	1.81 (1.22)	1.74 (1.25)	0.035	1.81 (1.22)	1.76 (1.19)	0.238
Myocardial infarction, *n* (%)	223 (15.31)	2206 (9.22)	<0.001	223 (15.31)	213 (14.62)	0.604
Congestive heart failure, *n* (%)	457 (31.37)	5176 (21.63)	<0.001	457 (31.37)	445 (30.54)	0.631
Peripheral vascular disease, *n* (%)	184 (12.63)	2472 (10.33)	0.005	184 (12.63)	173 (11.87)	0.534
Cerebrovascular disease, *n* (%)	198 (13.59)	3665 (15.32)	0.074	198 (13.59)	181 (12.42)	0.349
Dementia, *n* (%)	23 (1.58)	997 (4.17)	<0.001	23 (1.58)	40 (2.75)	0.030
Chronic respiratory disease, *n* (%)	372 (25.53)	5845 (24.43)	0.343	372 (25.53)	368 (25.26)	0.865
Type 2 diabetes mellitus, *n* (%)	298 (20.45)	5860 (24.49)	<0.001	298 (20.45)	298 (20.45)	0.999
Rheumatoid disease, *n* (%)	17 (1.17)	321 (1.34)	0.572	17 (1.17)	21 (1.44)	0.514
Peptic ulcer, *n* (%)	38 (2.61)	510 (2.13)	0.224	38 (2.61)	31 (2.13)	0.394
Mild/moderate/severe liver disease, *n* (%)	132 (9.06)	2620 (10.95)	0.024	132 (9.06)	123 (8.44)	0.555
Hemiplegia or paraplegia, *n* (%)	77 (5.28)	1502 (6.28)	0.128	77 (5.28)	73 (5.01)	0.737
Renal disease, *n* (%)	234 (16.06)	4050 (16.93)	0.391	234 (16.06)	205 (14.07)	0.133
Cancer and metastatic cancer, *n* (%)	381 (26.15)	6184 (25.85)	0.798	381 (26.15)	387 (26.56)	0.801
AIDS, *n* (%)	5 (0.34)	227 (0.95)	0.018	5 (0.34)	4 (0.27)	0.738
Underwent surgery, *n* (%)	1044 (71.65)	11,838 (49.48)	<0.001	1044 (71.65)	1048 (71.93)	0.869
Bronchial fibroscopy, *n* (%)	63 (4.32)	752 (3.14)	0.013	63 (4.32)	50 (3.43)	0.212
Non-invasive lung ventilation, *n* (%)	675 (46.33)	6738 (28.16)	<0.001	675 (46.33)	523 (35.90)	<0.001
Dialysis, *n* (%)	247 (16.95)	1618 (6.76)	<0.001	247 (16.95)	145 (9.95)	<0.001
Oxygen prior to hospitalization, *n* (%)	20 (1.37)	682 (2.85)	0.001	20 (1.37)	39 (2.68)	0.012
LOHS, median (IQR)	32 (38)	24 (28)	<0.001	32 (38)	25 (30)	<0.001
IHM, *n* (%)	519 (35.62)	6877 (28.74)	<0.001	519 (35.62)	444 (30.47)	0.003

POA: present on admission; HAP: hospital-acquired pneumonia; NV-HAP: non-ventilator hospital-acquired pneumonia; VAP: ventilator-associated pneumonia; SD: standard deviation; CCI: Charlson comorbidity index; AIDS: acquired immune deficiency syndrome; LOHS: length of hospital stay; IQR: interquartile range; IHM: in-hospital mortality.

## Data Availability

According to the contract signed with the Spanish Ministry of Health and Social Services, which provided access to the databases from the Spanish National Hospital Database (RAE-CMBD, *Registro de Actividad de Atención Especializada. Conjunto Mínimo Básico de Datos*, Registry of Specialized Health Care Activities, Minimum Basic Data Set), we cannot share the databases with any other investigator, and we must destroy the databases once the investigation has concluded. Consequently, we cannot upload the databases to any public repository. However, any investigator can apply for access to the databases by filling out the questionnaire available at http://www.msssi.gob.es/estadEstudios/estadisticas/estadisticas/estMinisterio/SolicitudCMBDdocs/Formulario_Peticion_Datos_CMBD.pdf (accessed on 4 January 2022). All other relevant data are included in the paper.

## Supplementary Materials

The following supporting information can be downloaded at: https://www.mdpi.com/article/10.3390/jcm11051179/s1, Table S1. International Classification of Diseases-10 (ICD-10) codes for diagnosis and therapeutic procedures used in this investigation. Table S2. Distribution of study covariates and hospital outcomes for people with hospital-acquired pneumonia in Spain (2016–2019), according to the presence of atrial fibrillation present prior to hospital admission or diagnosed during the hospitalization period. Table S3. Multivariable analysis of factors associated with in-hospital mortality during admission for hospital-acquired pneumonia among patients with of atrial fibrillation prior to hospital admission (POA = YES) according to sex. Table S4. Multivariable analysis of factors associated with in-hospital mortality during admission for hospital-acquired pneumonia among patients with atrial fibrillation diagnosed during the hospitalization (POA = NO) according to sex. Figure S1. Love plot for absolute standardized differences before and after propensity score matching (PSM) comparing covariate values for women with hospital-acquired pneumonia in Spain (2016–2019), according to the presence of atrial fibrillation diagnosed prior to hospital admission. Figure S2. Love plot for absolute standardized differences before and after propensity score matching (PSM) comparing covariate values for men with hospital-acquired pneumonia in Spain (2016–2019), according to the presence of atrial fibrillation diagnosed prior to hospital admission. Figure S3. Love plot for absolute standardized differences before and after propensity score matching (PSM) comparing covariate values for women with hospital-acquired pneumonia in Spain (2016–2019), according to the presence of atrial fibrillation diagnosed during the hospitalization period Figure S4. Love plot for absolute standardized differences before and after propensity score matching (PSM) comparing covariate values for men with hospital-acquired pneumonia in Spain (2016–2019), according to the presence of atrial fibrillation diagnosed during the hospitalization period
